# Predictive Values of N-Terminal Pro-B-Type Natriuretic Peptide and Cardiac Troponin I for Myocardial Fibrosis in Hypertrophic Obstructive Cardiomyopathy

**DOI:** 10.1371/journal.pone.0146572

**Published:** 2016-01-14

**Authors:** Changlin Zhang, Rong Liu, Jiansong Yuan, Jingang Cui, Fenghuan Hu, Weixian Yang, Yan Zhang, Youzhou Chen, Shubin Qiao

**Affiliations:** 1 State Key Laboratory of Cardiovascular Disease, Department of Cardiology, Fuwai Hospital, National Center for Cardiovascular Disease, Chinese Academy of Medical Sciences and Peking Union Medical College, Beijing, China; 2 State Key Laboratory of Cardiovascular Disease, Department of Radiology, Fuwai Hospital, National Center for Cardiovascular Disease, Chinese Academy of Medical Sciences and Peking Union Medical College, Beijing, China; Freeman Hospital and Newcastle University, UNITED KINGDOM

## Abstract

**Background:**

Both high-sensitivity cardiac troponin T and B-type natriuretic peptide are useful in detecting myocardial fibrosis, as determined by late gadolinium enhancement (LGE) cardiovascular magnetic resonance (CMR), in patients with non-obstructive hypertrophic cardiomyopathy. However, their values to predict myocardial fibrosis in hypertrophic obstructive cardiomyopathy (HOCM) remain unclear. We investigated the role of N-Terminal Pro-B-Type Natriuretic Peptide (NT-proBNP) and cardiac troponin I (cTnI) to identify LGE-CMR in patients with HOCM.

**Methods:**

Peripheral concentrations of NT-proBNP and cTnI were determined in patients with HOCM (n = 163; age = 47.2 ± 10.8 years; 38.7% females). Contrast-enhanced CMR was performed to identify and quantify myocardial fibrosis.

**Results:**

LGE was detected in 120 of 163 patients (73.6%). Patients with LGE had significantly higher levels of NT-proBNP and cTnI than those without LGE (1386.2 [904.6–2340.8] vs. 866.6 [707.2–1875.2] pmol/L, P = 0.003; 0.024 [0.010–0.049] vs. 0.010 [0.005–0.021] ng/ml, P <0.001, respectively). The extent of LGE was positively correlated with log cTnI (r = 0.371, P <0.001) and log NT-proBNP (r = 0.211, P = 0.007). On multivariable analysis, both log cTnI and maximum wall thickness (MWT) were independent predictors of the presence of LGE (OR = 3.193, P = 0.033; OR = 1.410, P < 0.001, respectively), whereas log NT-proBNP was not. According to the ROC curve analysis, combined measurements of MWT ≥21 mm and/or cTnI ≥0.025ng/ml indicated good diagnostic performance for the presence of LGE, with specificity of 95% or sensitivity of 88%.

**Conclusions:**

Serum cTnI is an independent predictor useful for identifying myocardial fibrosis, while plasma NT-proBNP is only associated with myocardial fibrosis on univariate analysis. Combined measurements of serum cTnI with MWT further improve its value in detecting myocardial fibrosis in patients with HOCM.

## Introduction

Hypertrophic cardiomyopathy (HCM) is a common inheritable cardiac disease with a prevalence of 1 in 500 of the general population [1, 2]. Asymmetric septal hypertrophy is the most common manifestation of this disorder, and ~70% of patients with HCM have associated left ventricular outflow tract (LVOT) obstruction, referred to as hypertrophic obstructive cardiomyopathy (HOCM) [3]. The presence of LVOT obstruction in HCM patients can not only lead to disabling symptoms of dyspnea, chest pain, and syncope, but also increases all-cause mortality and the occurrence of sudden cardiac death (SCD) in these patients [4].

Myocardial fibrosis, as a hallmark of HCM, can be identified noninvasively by contrast-enhanced cardiovascular magnetic resonance (CMR) imaging with late gadolinium enhancement (LGE), which has been demonstrated to be associated with SCD and other adverse outcomes [5–7].

Circulating N-Terminal Pro-B-Type Natriuretic Peptide (NT-proBNP) levels are elevated in patients with HCM, and correlate positively with symptoms of heart failure, hypertrophy severity, left ventricular diastolic dysfunction and LVOT gradient, and predict death and heart failure-related events [8–10]. Cardiac troponins are associated with extent of left ventricular hypertrophy, diastolic dysfunction, and adverse outcomes in patients with HCM [11–13].

Kawasaki et al reported that combined measurements of high-sensitivity cardiac troponin T (hs-cTnT) and B-Type Natriuretic Peptide (BNP) were useful for detecting myocardial fibrosis in patients with non-obstructive HCM [14]. Plasma levels of high-sensitivity C-reactive protein (hs-CRP) and endothlin-1 (ET-1) are elevated in patients with HCM, and levels of hs-CRP have been described to be associated with both histopathological myocardial fibrosis and LGE in CMR [15, 16]. However, those studies included a small number of patients with HCM, of whom the majority were non-obstructive HCM or non-obstructive HCM only. The associations between fibrosis as assessed by LGE, and circulating levels of NT-proBNP, cardiac troponin I (cTnI), big endothelin-1(big ET-1), hs-CRP or creatine kinase-MB isoenzyme (CK-MB) have not been established in patients with HOCM.

## Methods

### Study Population

We retrospectively included consecutive patients with HOCM who underwent a comprehensive cardiac evaluation in Fuwai Hospital (Beijing, China) between November 2008 and August 2013, by reviewing their de-identified medical records. The diagnosis of HCM was based on a maximum left ventricular wall thickness ≥15 mm (or ≥13 mm with an unequivocal family history of HCM), as measured by echocardiography or CMR, in the absence of any other accountable cardiac or systemic disease [17]. The presence of LVOT obstruction was defined as an instantaneous peak Doppler LVOT pressure gradient ≥30 mmHg at rest or during physiological provocation such as Valsalva manoeuvre, standing and exercise [17]. Patients with established coronary artery disease (prior myocardial infarction, ≥70% stenosis in any major epicardial coronary vessel on angiography, or previous coronary revascularization), valvular heart disease, left ventricular ejection fraction (LVEF) <50% as measured by echocardiography or CMR, renal dysfunction (defined as an estimated glomerular filtration rate <60 ml/min/1.73 m^2^), concomitant neoplasm, infection, or connective tissue disease were excluded. Subjects who had a history of alcohol septal ablation, septal myectomy or permanent mechanical device implantation were also excluded from the present study. Hypertension was defined as a systolic blood pressure ≥140mmHg and/or diastolic blood pressure ≥90mmHg, or receiving antihypertensive drugs.

The present study was performed in accordance with the Declaration of Helsinki. All procedures included in this study were undertaken as part of routine clinical practice, and the data which could identify subjects were removed. Informed consent was exempted because of the retrospective nature of the current study.

### Clinical and Echocardiographic Evaluation

All subjects underwent a comprehensive cardiac evaluation, including complete medical history, physical examination, 12-lead electrocardiography (ECG), 24-hour ambulatory ECG monitoring, transthoracic echocardiography (TTE), blood examination, CMR, and coronary angiography. Eventually, 163 patients were enrolled in the present study. TTE was performed using the Phillips iE33 Color Doppler Ultrasound System (Philips Healthcare, Andover, MA, USA). M-mode, 2-dimensional, and pulsed and continuous-wave Doppler study were used in the standard evaluation in accordance with recommendations of the American Society of Echocardiography [18]. The severity of mitral regurgitation (MR), which was graded from mild to severe according to EAE criteria, was evaluated semiquantitatively with Color Doppler flow imaging [19].

### CMR Image Acquisition

All CMR studies were performed with a 1.5-Tesla scanner (Magnetom Avanto, Siemens Medical Solutions, Erlangen, Germany), under breath control and electrocardiographic gating. The CMR imaging protocol was reported previously [20]. A true fast imaging with steady-state precession (TrueFisp) sequence was used to obtain cine images, which included left ventricular (LV) 2-chamber and 4-chamber long-axis view, LVOT view and LV short-axis views (contiguous slices from base to apex for full coverage of the LV). Typical imaging parameters were as follows: field of view (FOV) 360×315 mm^2^, slice thickness 6 mm, inter-slice gap 2 mm, at least 20 phases, repetition time (TR) 2.7 ms, echo time (TE) 1.2 ms, temporal resolution 40 ms, image matrix 192×162, and flip angle 70°. Images of LGE were acquired 10–15 minutes after bolus injection of 0.2 mmol/kg gadolinium-diethylenetriamine pentaacetic acid (Gd-DTPA, Magnevist, Schering AG, Berlin, Germany), using a segmented phase-sensitive inversion recovery (PSIR) Turbo Fast Low Angle Shot (FLASH) sequence in the same views as used in cine imaging. The optimal inversion time was adjusted to null normal myocardium typically between 250 and 350 ms. Typical imaging parameters were: FOV 380×320 mm^2^, slice thickness 6 mm, inter-slice gap 2 mm, TR 8.6 ms, TE 3.36 ms, image matrix 256×162, flip angle 25°, pixel size 2×1.5 mm^2^, and parallel acquisition technique (PAT) factor of 2.

### Image Analysis

All CMR images were transferred to workstation (Siemens Medical Systems, Erlangen, Germany). The dedicated software (version VE36A, ARGUS, Siemens, Germany) was used for image analysis off-line.

Endocardial and epicardial contours of the LV myocardium (excluding papillary muscles) were manually traced at end diastole and end systole on each LV short-axis cine image. LV end-diastolic volume (LVEDV), LV end-systolic volume (LVESV), LV ejection fraction (LVEF), stroke volume (SV), cardiac output (CO), and LV mass (LVM) were then calculated in a standard fashion. LV mass was derived by multiplying LV myocardial volume measured at end diastole with the specific gravity of myocardium (1.05 g/ml). Furthermore, all of those parameters were indexed to body surface area (BAS), except LVEF. The maximal septal and LV wall thickness were traced and measured from the short-axis views at end diastole. The left atrial diameter was determined as the distance from the center of the mitral annulus to the posterior atrial wall on vertical and horizontal long-axis cine images.

**Fig 1:**
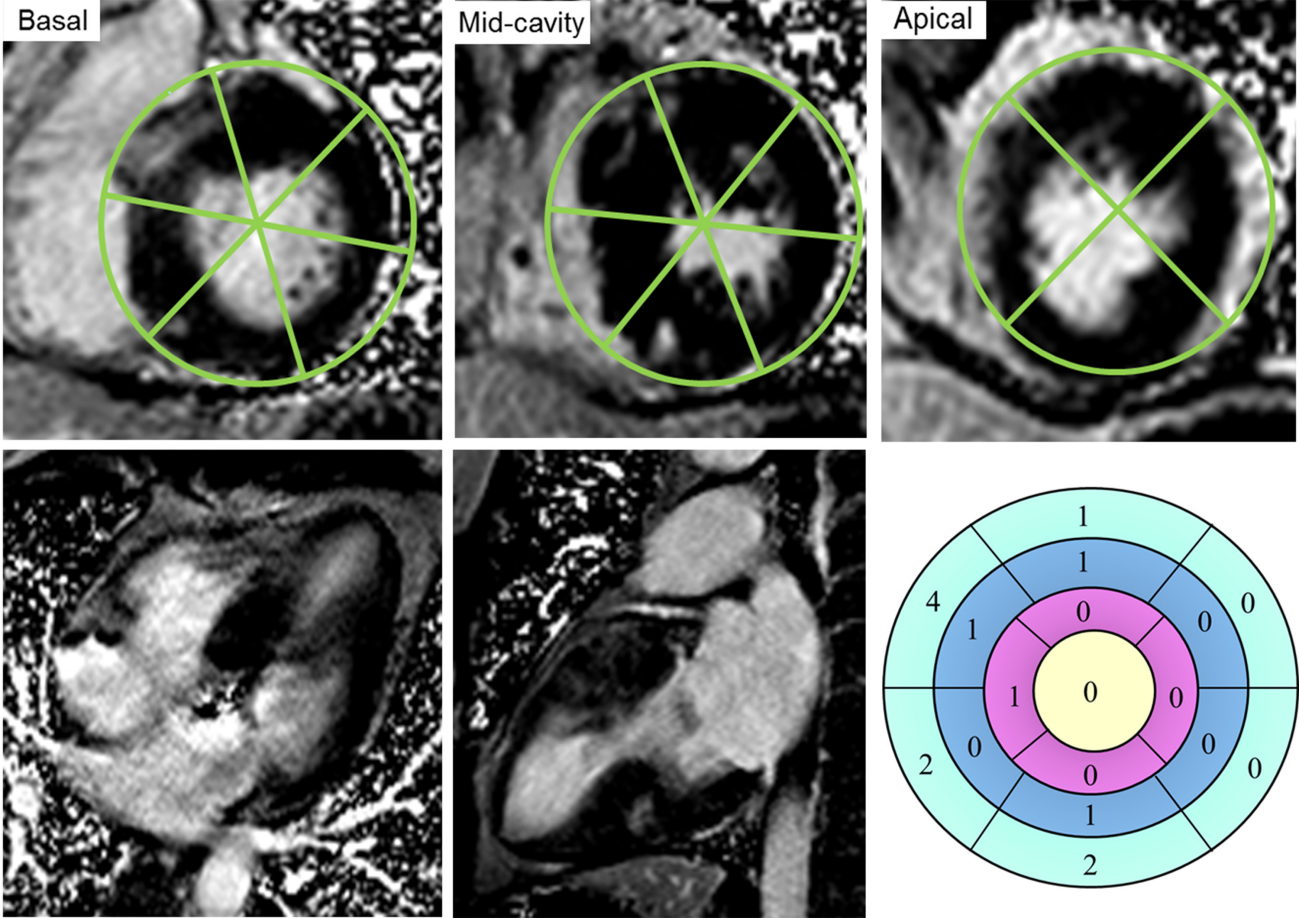
A schematic diagram of the quantification of LGE by the visual scoring method based on the standard 17-segment model of the left ventricle. The number in each segment represents the corresponding LGE score of that segment. By adding each individual segment score, the total LGE score was 13 for this patient. The extent of LGE (LGE %) was then calculated as 19.1% ([13/68]×100). Details of the scoring procedure are described in the Methods section. LGE indicates late gadolinium enhancement.

LGE was estimated both qualitatively as a binomial variable (positive/negative) and quantitatively. The presence of any higher signal intensity area compared to the nulled normal myocardium was determined as LGE positive, otherwise LGE negative, by reviewing all short and long axis contrast-enhanced artefact-free images. The quantification of LGE was performed using the previously described visual scoring method based on the standard 17-segment model of the left ventricle [21, 22]. The extent of hyperenhancement within each segment was visually evaluated and scored as 0 (no enhancement), 1 (0–25% enhancement), 2 (26–50% enhancement), 3 (51–75% enhancement) or 4 (76–100% enhancement). Segment 1–16 were analyzed on three representative short-axis slices obtained at the base (at the mitral valve), mid-ventricle (at the papillary muscles), and apex (below the papillary muscles) which showed visually the greatest amount of LGE. Specifically, the 17th segment was the true apex and analyzed on the vertical and horizontal long-axis views. Summing the individual score of LGE in each of 17 segments yielded the global volume of LGE (LGE score), which was thereafter expressed as a percentage of the total maximum score (4 × 17 = 68): 100 × (LGE score) / 68 (Fig 1).

From short axis planes, the location of LGE in the LV were recorded as: (1) right ventricular insertion points (the anterior and inferior attachment points of the right ventricle to the interventricular septum); (2) septum (all other septal hyperenhancements); (3) apical (confined to the LV apex); and (4) all other LV locations [7].

### Laboratory Measurements

Venous blood samples were drawn under resting conditions within 2 days of TTE and 7 days of CMR examination. All of the following biomarkers were measured within 4 hours of blood collection as routine sample analysis.

Plasma levels of NT-proBNP were measured using an electrochemiluminescent immunoassay (Elecsys proBNP II assay, Roche Diagnostics GmbH, Mannheim, Germany) on a Cobas 6000 analyzer (Roche Diagnostics), with a lower detection limit of 0.6 pmol/L. The inter-assay coefficient of variation was ≤4.6% and the intra-assay coefficient of variation was ≤4.2%.

For measurement of serum cTnI, an immunochemiluminometric assay (Access AccuTnI, Beckman Coulter, CA, USA) was performed on a Beckman Coulter Access 2 analyzer. According to the manufacturer, the lower limit of detection is 0.01ng/ml. The upper limit of normal (the 99th percentile of normal population) was 0.04ng/ml. The inter-assay and intra-assay coefficients of variation were less than 6.07% and 4.42%, respectively.

Circulating endothelin (ET) has a very short half-life, while its precursor (big ET-1) has a longer half-life and is easier to be detected. Plasma big ET-1 was measured using a commercial enzyme immunoassay (Bl-20082H big Endothelin-1, Biomedica, Wien, Austria) on a Cobas 6000 analyzer (Roche Diagnostics), with a detection limit of 0.02 pmol/L. Cross-reactivity with ET-1, ET-2, and ET-3 was less than 1%. The inter-assay and intra-assay coefficients of variation were less than 4% and 5%, respectively.

Serum hs-CRP was measured using the Particle Enhanced Immunoturbidimetric Assay (Ultrasensitive CRP kit, Orion Diagnostica, Espoo, Finland) on an Olympus AU-5400 analyzer (Olympus Diagnostics), with a lower detection limit of 0.25 mg/L. The inter-assay coefficient of variation was ≤11.9% and the intra-assay coefficient of variation was ≤6.6%.

Serum CK-MB was determined by an immunoinhibition assay (creatine kinase-MB kit, Biosino, Beijing, China) on an Olympus AU-5400 analyzer (Olympus Diagnostics), with a lower detection limit of 3 U/L. The inter-assay and intra-assay coefficients of variation were less than 6%.

### Statistical Analysis

Statistical analysis was performed with the statistical package SPSS 21.0 (SPSS Inc, Chicago, IL, USA). A 2-tailed P value of <0.05 was considered to be statistically significant. Continuous variables are expressed as mean ± standard deviation (SD), or median (interquartile range [IQR]), and were compared with the independent-samples t-test or the Mann–Whitney U-test (as appropriate). The comparison of categorical variables, which were expressed as frequencies (percentages), was performed using χ^2^ test or Fisher’s exact test (as appropriate). Correlation between two continuous variables was examined using Pearson’s correlation test or Spearman’s correlation test (as appropriate). As the values of NT-proBNP, cTnI, and big-ET follow lognormal distribution, they were subjected to logarithmic transformations for t tests, scatterplots, correlations and regression analysis.

Multivariate binary logistic regression analysis using backward elimination was performed to identify potential predictors of the presence of LGE. The predicting variables were pre-specified on the basis of univariate p values of <0.10 and previously published literature. The included variables were as follows: age, hypertension, duration from initial diagnosis, family history of HCM, New York Heart Association (NYHA) functional class, calcium channel antagonists, resting LVOT gradient, LVEF, left ventricular end-diastolic volume index (LVEDVI), left ventricular mass index (LVMI), maximum wall thickness (MWT), log NT-proBNP, log cTnI, and hs-CRP. The optimal cut-off values for cTnI and MWT to predict LGE were determined by receiver-operating characteristic (ROC) curve analysis. Sensitivity, specificity and positive and negative predictive values were calculated for selected cTnI and MWT cut-off points.

## Results

### Clinical Characteristics

One hundred and sixty three patients with HOCM were included in this study (100 men; mean age 47.2 ± 10.8, range 20 to 71 years). Baseline characteristics are presented in Table 1. Median duration from initial diagnosis of HOCM to the study enrollment was 12 (interquartile range [IQR] 2–48) months. The majority of this cohort experienced dyspnea (89.6%), and were of New York Heart Association functional class III/IV (69.9% / 16.0%), although all patients had normal left ventricular systolic function, as in accordance with the exclusion criteria. Of the 163 patients with HOCM, 76.7% were receiving β-blockers, and 31.3% calcium antagonists. All participants had LVOT obstruction (LVOT gradient at rest, 78.7 ± 32.5 mmHg), including 11 (6.7%) patients with latent LVOT obstruction in whom the obstruction was only present after provocation (Table 2).

**Table 1:** Baseline characteristics of patients with and without LGE.

Variable	Overall population	LGE-positive	LGE-negative	P Value
	(n = 163)	(n = 120)	(n = 43)	
Age, years	47.2 ± 10.8	45.5 ± 10.6	52.0 ± 10.0	0.001
Men, n(%)	100 (61.3%)	78 (65.0%)	22 (51.2%)	0.110
Body surface area, m^2^	1.78 ± 0.19	1.78 ± 0.19	1.76 ± 0.17	0.560
Hypertension, n (%)	48 (29.4%)	31 (25.8%)	17 (39.5%)	0.090
Diabetes mellitus, n (%)	6 (3.7%)	5 (4.2%)	1 (2.3%)	0.938
Hypercholesterolemia, n (%)	47 (28.8%)	31 (25.8%)	16 (37.2%)	0.160
Current smokers, n (%)	59 (36.2%)	46 (38.3%)	13 (30.2%)	0.340
Duration from diagnosis, median (IQR), months	12 (2–48)	12 (3–48)	4 (1–48)	0.027
Family history of HCM, n (%)	37 (22.7%)	32 (26.7%)	5 (11.6%)	0.043
Family history of sudden death, n (%)	18 (11.0%)	15 (12.5%)	3 (7.0%)	0.479
Dyspnea, n (%)	146 (89.6%)	108 (90.0%)	38 (88.4%)	0.993
NYHA functional class				0.050
I	9 (5.5%)	7 (5.8%)	2 (4.7%)	
II	14 (8.6%)	13 (10.8%)	1 (2.3%)	
III	114 (69.9%)	84 (70%)	30 (69.8%)	
IV	26 (16.0%)	16 (13.3%)	10 (23.3%)	
Chest pain, n (%)	57 (35.0%)	38 (31.7%)	19 (44.2%)	0.140
Syncope, n (%)	54 (33.1%)	38 (31.7%)	16 (37.2%)	0.508
Palpitation, n (%)	30 (18.4%)	23 (19.2%)	7 (16.3%)	0.675
Atrial fibrillation, n (%)	14 (8.6%)	11 (9.2%)	3 (7.0%)	0.902
Non-sustained VT*, n (%)	9/127 (7.1%)	7/95 (7.4%)	2/32 (6.3%)	1
Systolic blood pressure, mmHg	117.2 ± 17.4	116.6 ± 17.3	118.8 ± 17.7	0.476
Diastolic blood pressure, mmHg	72.9 ± 11.1	73.5 ± 11.2	71.3 ± 10.9	0.283
Heart rate (beats/min)	69.4 ± 11.0	69.4 ± 11.1	69.4 ± 11.1	0.997
Medications, n (%)				
Beta-blockers	125 (76.7%)	92 (76.7%)	33 (76.7%)	0.992
Calcium antagonists	51 (31.3%)	31 (25.8%)	20 (46.5%)	0.012
Amiodarone	8 (4.9%)	8 (6.7%)	0 (0.0%)	0.185
ACEI/ARB	25 (15.3%)	16 (13.3%)	9 (20.9%)	0.236
Statins	18 (11.0%)	11 (9.2%)	7 (16.3%)	0.321
Aspirin	40 (24.5%)	27 (22.5%)	13 (30.2%)	0.312
Diuretics	9 (5.5%)	8 (6.7%)	1 (2.3%)	0.496
Trimetazidine	6 (3.7%)	6 (5.0%)	0 (0.0%)	0.307

ACEI indicates angiotensin-converting enzyme inhibitor; ARB, angiotensin receptor blocker; HCM, hypertrophic cardiomyopathy; IQR, interquartile range; LGE, late gadolinium enhancement; NYHA, New York Heart Association, and VT, ventricular tachycardia.

*Holter monitoring results were available in 127 of 163 study patients.

**Table 2:** Echocardiography and CMR data of patients with and without LGE.

Variable	Overall population	LGE-positive	LGE-negative	p Value
	(n = 163)	(n = 120)	(n = 43)	
Echocardiography				
SAM, n (%)	154 (94.5%)	113 (94.2%)	41(95.3%)	1.000
MR, n (%)				0.650
Absent	16(9.8%)	12(10.0%)	4(9.3%)	
Mild	85(52.1%)	64(53.3%)	21(48.8%)	
Moderate	56(34.4%)	39(32.5%)	17(39.5%)	
Severe	6(3.7%)	5(4.2%)	1(2.3%)	
LVOTG at rest, mmHg	78.7 ± 32.5	74.2 ± 28.0	91.2 ± 40.2	0.003
Peak LVOTG (at rest or after provocation), mmHg *	91.1 ± 26.0	87.5 ± 23.7	101.1 ± 29.5	0.003
LVOTG after provocation, mmHg *	93.2 ± 25.5 (n = 50)	92.7 ± 25.7 (n = 39)	94.8 ± 26.0 (n = 11)	0.812
Latent LVOT obstruction, n (%)	11 (6.7%)	6 (6.0%)	5 (11.6%)	0.258
Concomitant mid-ventricular obstruction, n (%)	9 (5.5%)	9 (7.5%)	0 (0.0%)	0.145
CMR				
LAD, mm	40.2 ± 7.7	40.2 ± 7.9	40.0 ± 7.2	0.865
LVEDD, mm	45.7 ± 4.3	45.6 ± 4.1	46.1 ± 4.9	0.575
MWT, mm	23.3 ± 4.8	24.3 ± 4.9	20.4 ± 2.5	<0.001
MWT≥30mm, n (%)	14(8.6%)	14 (11.7%)	0 (0%)	0.043
LVEF, %	72.1 ± 7.2	70.9 ± 7.2	75.6 ± 5.8	<0.001
LVEDVI, ml/m^2^	64.7 ± 14.0	66.0 ± 14.1	61.1 ± 13.2	0.048
LVESVI, ml/m^2^	18.1 ± 6.5	19.3 ± 6.5	14.9 ± 5.2	<0.001
SVI, ml/m^2^	46.6 ± 10.7	46.7 ± 10.9	46.2 ± 10.2	0.788
CI, L/min/m^2^	3.12 ± 0.78	3.13 ± 0.78	3.10 ± 0.78	0.859
LVMI, g/m^2^	91.7 ± 32.6	97.7 ± 33.0	75.0 ± 24.7	<0.001
LGE Score	15.8 ± 12.7	21.5 ± 9.8	0 ± 0	NA
LGE %	23.2 ± 12.7	31.6 ± 14.5	0 ± 0	NA
Location of LGE, n (%)				
RV insertion points	109 (66.9%)	109 (90.8%)	0 (0%)	NA
other septal locations	60 (36.8%)	60 (50.0%)	0 (0%)	NA
LGE-LV apex	22 (13.5%)	22 (18.3%)	0 (0%)	NA
LGE-other locations	34 (20.9%)	34 (28.3%)	0 (0%)	NA

CI indicates cardiac index; CMR, cardiac magnetic resonance; LAD, left atrium diameter; LGE, late gadolinium enhancement; LV, left ventricle; LVEDD, left ventricular end-diastolic diameter; LVEDVI, left ventricular end-diastolic volume index; LVEF, left ventricular ejection fraction; LVESVI, left ventricular end-systolic volume index; LVMI, left ventricular myocardium mass index; LVOT, left ventricular outflow tract; LVOTG, left ventricular outflow tract gradient; MR, mitral regurgitation; MWT, maximum wall thickness; NA, not applicable; SAM, systolic anterior motion; and SVI, stroke volume index.

*Provoked LVOT gradient results were obtained in 50 of 163 study patients.

LGE was identified in 120 of 163 patients (73.6%). Patients with LGE were younger (45.5 ± 10.6 vs. 52.0 ± 10.0 years, P = 0.001), and were more likely to have a family history of HCM (26.7% vs. 11.6%, P = 0.043), than those without LGE. The median duration from initial diagnosis of HOCM to study entry was longer in patients with LGE compared to patients without LGE (12 [IQR, 3–48] vs. 4 [IQR, 1–48] months, P = 0.027). There was a trend towards more NYHA functional class II and less NYHA functional class IV in patients with LGE (P = 0.05). Medications taken did not differ significantly between the two groups, except that patients with LGE were less likely to take calcium antagonists (25.8% vs. 46.5%, P = 0.012). No other significant differences were observed between LGE-positive group and LGE-negative group, concerning family history of sudden death, history of syncope, non-sustained ventricular tachycardia or atrial fibrillation.

### Echocardiographic and CMR Parameters in Relation to the Presence and Extent of LGE in HOCM Patients

Table 2 shows the echocardiographic and CMR parameters in patients with and without LGE. Compared with LGE-negative patients, LGE-positive patients had lower resting LVOT gradient (74.2 ± 28.0 vs. 91.2 ± 40.2 mmHg, P = 0.003), and peak LVOT gradient (at rest or after provocation, 87.5 ± 23.7 vs. 101.1 ± 29.5 mmHg, P = 0.003). There were no significant differences with respect to the prevalence of latent LVOT obstruction (6.0% vs. 11.6%, P = 0.258), systolic anterior motion (SAM) (94.2% vs. 95.3%, P = 1.000), or concomitant mid-ventricular obstruction (7.5% vs. 0.0%, P = 0.145), or the severity of mitral regurgitation (MR), between the two groups.

Patients with LGE had greater maximum wall thickness (24.3 ± 4.9 vs. 20.4 ± 2.5 mm, P <0.001) and LVMI (97.7 ± 33.0 vs. 75.0 ± 24.7 g/m^2^, P <0.001), and also higher proportion of maximum wall thickness ≥30mm (11.7% vs. 0%, P = 0.043), as compared to those without LGE. LVEDVI and left ventricular end-systolic volume index (LVESVI) were larger in LGE-positive patients than in LGE-negative patients (66.0 ± 14.1 vs. 61.1 ± 13.2 ml/m^2^, P = 0.048; 19.3 ± 6.5 vs. 14.9 ± 5.2 ml/m^2^, P <0.001; respectively). In contrast, left ventricular ejection fraction (LVEF) was lower in patients with LGE than in those without LGE (70.9 ± 7.2% vs. 75.6 ± 5.8%, P <0.001), although all patients had normal systolic function (LVEF ≥50%).

Within the LGE-positive group, the extent of LGE (presented as LGE%) involved 31.6 ± 14.5% of left ventricular myocardium ranging from 2.9% to 73.5%. The most frequently observed locations for LGE were the RV insertion points identified in 109 of 120 patients (90.8%), followed by other septal locations in 60 (50.0%), other LV locations in 34 (28.3%) and the LV apex in 22 (18.3%) patients.

**Fig 2:**
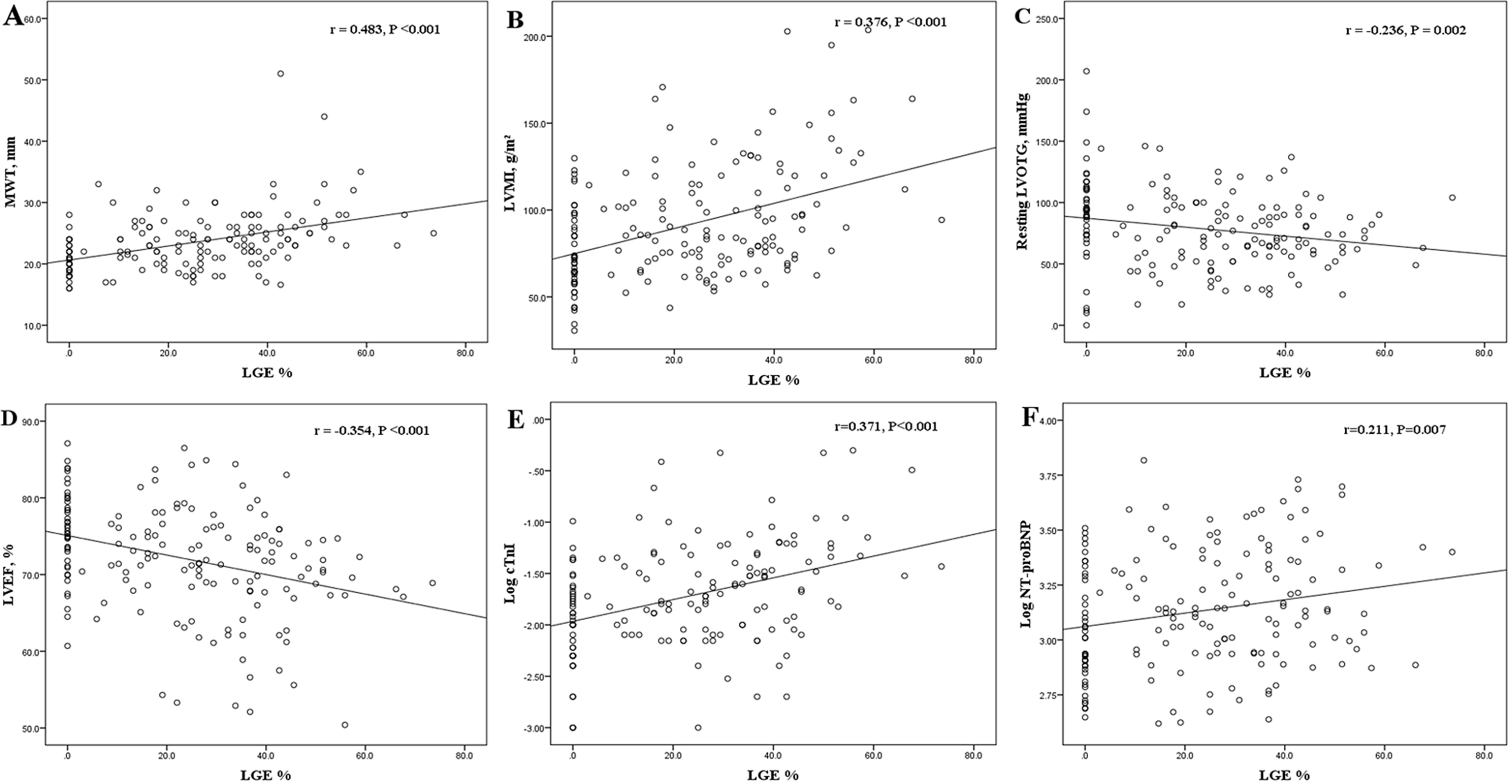
Correlations between the extent of LGE (LGE%) and MWT (A), LVMI (B), resting LVOTG (C), LVEF (D), Log cTnI (E) and log NT-proBNP (F) in the overall study patients. cTnI indicates cardiac troponin I; LVEF, left ventricular ejection fraction; LVMI, left ventricular mass index; LVOTG, left ventricular outflow tract gradient; MWT, maximum wall thickness; NT-proBNP, N-terminal pro B-type natriuretic peptide. Other abbreviations as in Fig 1.

Among the overall studied population, the extent of LGE was positively correlated with maximum wall thickness (r = 0.483, P <0.001), LVESVI (r = 0.303, P <0.001), and LVMI (r = 0.376, P <0.001); in contrast, it was inversely correlated with age (r = -0.289, P <0.001), resting LVOT gradient (r = -0.236, P = 0.002), peak LVOT gradient (r = -0.274, P <0.001), and LVEF (r = -0.354, P <0.001) (Fig 2 and Table 3). In patients with LGE, LGE% was directly related to maximum wall thickness (r = 0.292, P = 0.001) and LVMI (r = 0.320, P <0.001), and was inversely related to peak LVOT gradient (r = -0.194, P = 0.033) and LVEF (r = -0.195, P = 0.033). However, in the LGE-positive group, there were no significant correlations between LGE% and age (r = -0.169, P = 0.065), resting LVOT gradient (r = -0.076, P = 0.407), and LVESVI (r = 0.106, P = 0.251). In addition, no significant correlations were identified between LGE% and duration from diagnosis to study entry, NYHA functional class or LVEDVI in the overall cohort or LGE-positive cohort.

**Table 3:** Correlates of extent of LGE (LGE %) in the overall patients and patients with LGE.

Variable	Overall population		LGE-positive	
	(n = 163)		(n = 120)	
LGE %	r	P value	r	P value
Age, years	-0.289	<0.001	-0.169	0.065
BMI, kg/m^2^	-0.086	0.274	0.043	0.644
BSA, m^2^	0.158	0.044	0.254	0.005
Duration from diagnosis, months	0.076	0.338	-0.115	0.213
Log NT-proBNP	0.211	0.007	0.068	0.462
Log cTnI	0.371	<0.001	0.235	0.013
Resting LVOTG, mmHg	-0.236	0.002	-0.076	0.407
Peak LVOTG, mmHg	-0.274	<0.001	-0.194	0.033
LAD, mm	0.005	0.948	0.009	0.922
MWT, mm	0.483	<0.001	0.292	0.001
LVEF, %	-0.354	<0.001	-0.195	0.033
LVEDVI, ml/m^2^	0.110	0.161	-0.045	0.629
LVESVI, ml/m^2^	0.303	<0.001	0.106	0.251
LVMI, g/m^2^	0.376	<0.001	0.320	<0.001

BMI indicates body mass index; BSA, body surface area; cTnI, cardiac troponin I; LAD, left atrium diameter; LGE, late gadolinium enhancement; LVEDVI, left ventricular end-diastolic volume index; LVEF, left ventricular ejection fraction; LVESVI, left ventricular end-systolic volume index; LVMI, left ventricular myocardium mass index; LVOTG, left ventricular outflow tract gradient; MWT, maximum wall thickness; and NT-proBNP, N-terminal pro B-type natriuretic peptide.

### Relationship between Levels of Circulating Biomarkers and the Presence and Extent of LGE

**Fig 3:**
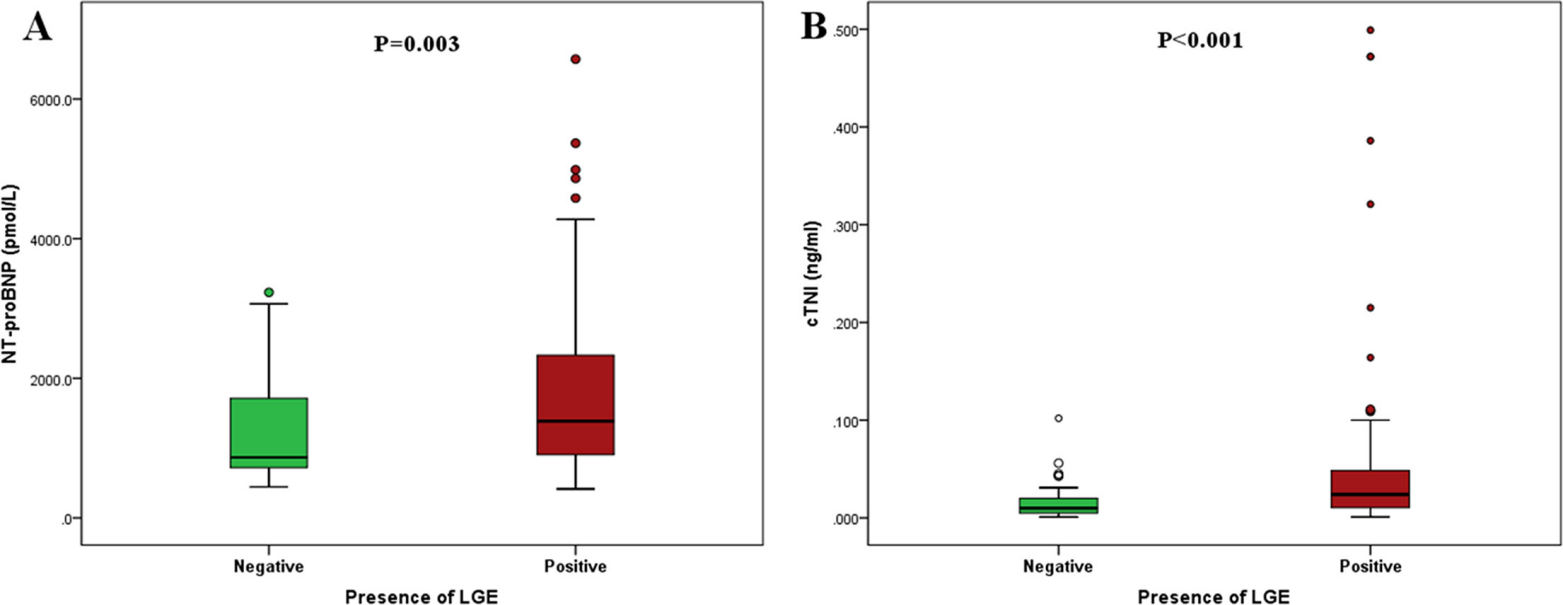
Concentrations (medians and interquartile ranges) of NT-proBNP (A) and cTnI (B) in patients with and without LGE.

The median levels of NT-proBNP and cTnI were significantly higher in patients with LGE than in those without LGE (Fig 3; 1386.2 [IQR 904.6–2340.8] vs. 866.6 [IQR 707.2–1875.2] pmol/L, P = 0.003; 0.024 [IQR 0.010–0.049] vs. 0.010 [IQR 0.005–0.021] ng/ml, P <0.001, respectively). Additionally, the values of hs-CRP, big endothelin-1, and CK-MB were comparable between patients with and without LGE (Table 4).

**Table 4:** Levels of circulating biomarkers in patients with and without LGE^*^.

Variable	Overall population	LGE-positive	LGE-negative	p Value
	(n = 163)	(n = 120)	(n = 43)	
NT-proBNP, pmol/L	1314.9 (860.6–2233.4)	1386.2 (904.6–2340.8)	866.6 (707.2–1875.2)	0.003
Log NT-proBNP	3.13 ± 0.27	3.17 ± 0.27	3.03 ± 0.25	0.003
cTnI, ng/ml	0.019 (0.008–0.043)	0.024 (0.010–0.049)	0.010 (0.005–0.021)	<0.001
Log cTnI	-1.72 ± 0.52	-1.61 ± 0.50	-2.01 ± 0.48	<0.001
Big ET-1, pmol/L	0.56 (0.41–0.74)	0.57 (0.38–0.73)	0.52 (0.41–0.76)	0.898
Log big ET-1	0.19 ± 0.07	0.19 ± 0.08	0.20 ± 0.07	0.762
hs-CRP (mg/L)	1.23 (0.55–2.02)	1.10 (0.53–1.87)	1.59 (0.60–2.61)	0.074
CK-MB (IU/L)	11 (9–13)	12 (9–13)	10 (8–14)	0.249

Big ET-1 indicates big endothelin-1; CK-MB, creatine kinase-MB isoenzyme; cTnI, cardiac troponin I; hs-CRP, high-sensitivity C-reactive protein; LGE, late gadolinium enhancement; and NT-proBNP, N-terminal pro B-type natriuretic peptide.

*Values are mean ± SD or median (IQR) as appropriate.

**Fig 4:**
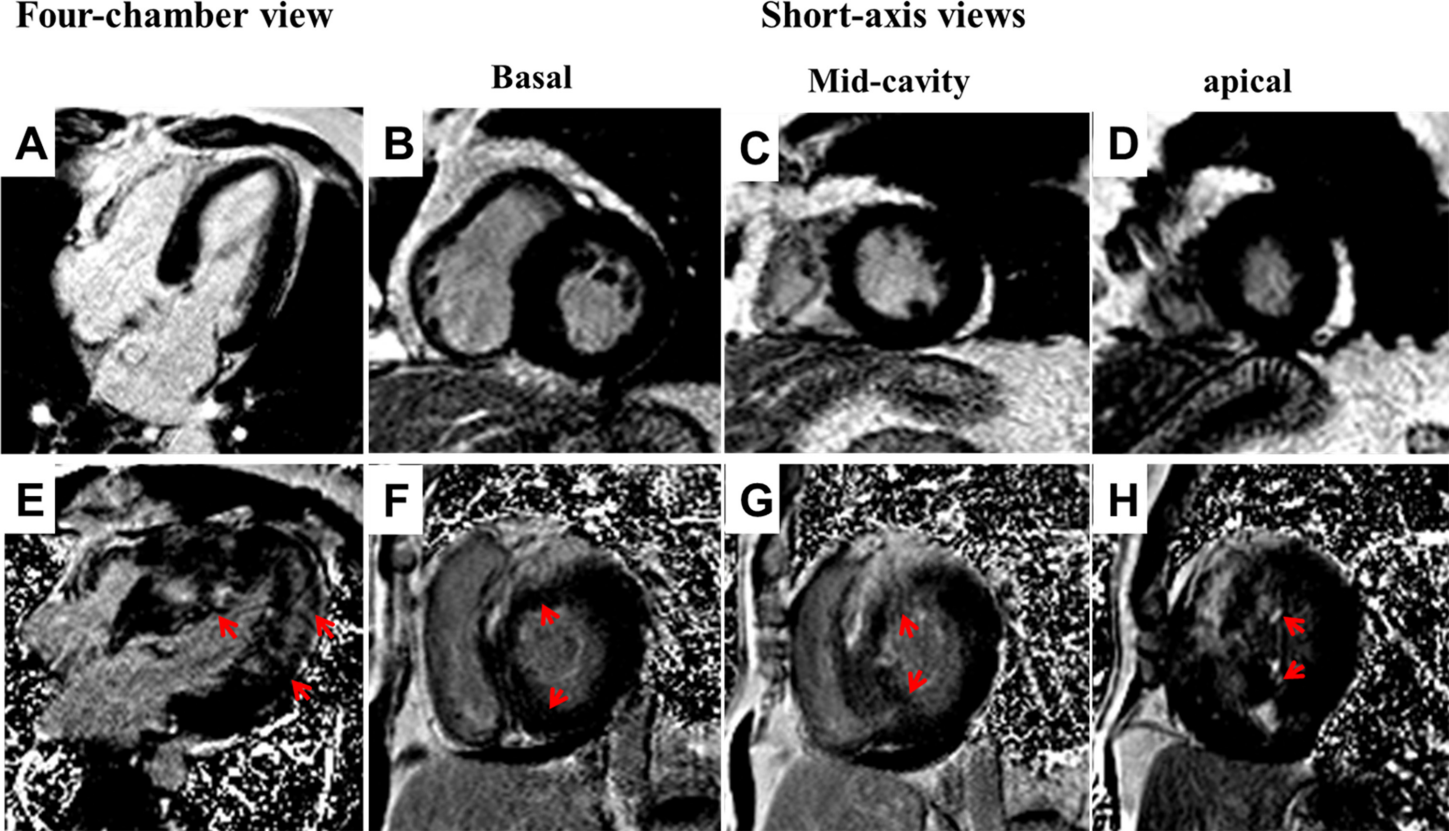
Representative LGE images of patients with extensive and without LGE. A 61-year-old woman with slightly elevated NT-proBNP plasma level (515.4pmol/L) and normal serum level of cTnI (0.006ng/ml), had no LGE detected in the 4-chamber view and end-diastolic short-axis views at basal, mid-ventricular and apical levels of left ventricle (A-D). A 47-year-old man with significantly elevated peripheral levels of NT-proBNP (2642.0pmol/L) and cTnI (0.321ng/ml), had extensive LGE (red arrows; LGE score, 46; LGE%, 67.6%) mainly involving the anterior, anteroseptal, septal, inferoseptal and inferior myocardium (E-H). Abbreviations as in Figs 1 and 2.

LGE% was positively correlated with log cTnI (r = 0.371, P <0.001) and log NT-proBNP (r = 0.211, P = 0.007) in the overall patients with HOCM (Fig 2 and Table 3). In patients with LGE, the extent of LGE was significantly correlated with log cTnI (r = 0.235, P = 0.013), whereas it did not correlate with log NT-proBNP (r = 0.068, P = 0.462). Representative CMR images of two patients demonstrating the relationship between the extent of LGE and the peripheral levels of NT-proBNP and cTnI are shown in Fig 4. In the overall cohort, LGE% had no correlations with hsCRP (r = -0.149, P = 0.058) and log big ET-1 (r = 0.076, P = 0.336), although it was slightly corelated with the level of CK-MB (r = 0.156, P = 0.047). No correlations were found between LGE% and log big ET-1 (r = 0.150, P = 0.102), hsCRP (r = -0.079, P = 0.393), and CK-MB (r = 0.170, P = 0.064) in the LGE-positive patients.

Log NT-proBNP was significantly associated with log cTnI in the overall patients with HOCM (r = 0.270, P = 0.001) and in patients with LGE (r = 0.212, P = 0.026), but not in patients without LGE (r = 0.220, P = 0.166) (Table 5). In addition, log NT-proBNP was significantly correlated with resting LVOT gradient (r = 0.226, P = 0.004), left atrium diameter (LAD) (r = 0.298, P < 0.001), maximum wall thickness (r = 0.358, P < 0.001), and LVMI (r = 0.456, P < 0.001), and had similar correlations with these parameters in patients with and without LGE, except no correlation with MWT in the LGE-negative patients. The level of cTnI was positively correlated with maximum wall thickness (r = 0.437, P <0.001) and LVMI (r = 0.497, P <0.001) in the overall cohort. Similar correlations were observed in patients with and without LGE, besides no significant correlation between log cTnI and MWT in patients without LGE. Additionally, log cTnI was significantly correlated with LVEDVI (r = 0.201, P = 0.013), LVESVI (r = 0.246, P = 0.002), and LVEF (r = -0.179, P = 0.027) in the overall cohort. It also had positive correlations with LVEDVI (r = 0.368, P = 0.018) and LVESVI (r = 0.371, P = 0.017) in patients without LGE.

**Table 5:** Correlates of peripheral levels of NT-proBNP and cTNI in patients With HOCM.

Variable	Overall population		LGE-positive		LGE-negative	
	(n = 163)		(n = 120)		(n = 43)	
	r	P value	r	P value	r	P Value
Log NT-pro BNP						
Age, years	0.041	0.605	0.137	0.135	0.014	0.931
BMI, kg/m^2^	-0.304	<0.001	-0.297	0.001	-0.279	0.070
BSA, m^2^	-0.347	<0.001	-0.386	<0.001	-0.306	0.046
Duration from diagnosis, months	0.139	0.076	0.095	0.304	0.122	0.435
Log NT-proBNP	NA		NA		NA	
Log cTnI	0.270	0.001	0.212	0.026	0.220	0.166
Resting LVOTG, mmHg	0.226	0.004	0.203	0.026	0.512	<0.001
Peak LVOTG, mmHg	0.041	0.605	-0.006	0.946	0.362	0.017
LAD, mm	0.298	<0.001	0.303	0.001	0.306	0.046
MWT, mm	0.358	<0.001	0.318	<0.001	0.254	0.100
LVEF, %	-0.069	0.384	-0.041	0.658	0.149	0.341
LVEDVI, ml/m^2^	0.137	0.081	0.045	0.625	0.305	0.047
LVESVI, ml/m^2^	0.118	0.132	0.047	0.608	0.078	0.619
LVMI, g/m^2^	0.456	<0.001	0.386	<0.001	0.549	<0.001
Log cTnI						
Age, years	-0.061	0.456	0.025	0.792	0.068	0.673
BMI, kg/m^2^	-0.044	0.591	0.088	0.357	-0.352	0.024
BSA, m^2^	0.074	0.368	0.151	0.115	-0.274	0.082
Duration from diagnosis, months	0.030	0.715	-0.017	0.862	-0.045	0.780
Log NT-proBNP	0.270	0.001	0.212	0.026	0.220	0.166
Log cTnI	NA		NA		NA	
Resting LVOTG, mmHg	-0.070	0.391	-0.109	0.255	0.296	0.060
Peak LVOTG, mmHg	-0.158	0.051	-0.143	0.136	0.065	0.686
LAD, mm	0.095	0.245	0.081	0.396	0.153	0.338
MWT, mm	0.437	<0.001	0.410	<0.001	0.115	0.476
LVEF, %	-0.179	0.027	-0.038	0.689	-0.238	0.133
LVEDVI, ml/m^2^	0.201	0.013	0.084	0.383	0.368	0.018
LVESVI, ml/m^2^	0.246	0.002	0.092	0.335	0.371	0.017
LVMI, g/m^2^	0.497	<0.001	0.437	<0.001	0.467	0.002

BMI indicates body mass index; BSA, body surface area; cTnI, cardiac troponin I; HOCM, hypertrophic obstructive cardiomyopathy; LAD, left atrium diameter; LVEDVI, left ventricular end-diastolic volume index; LVEF, left ventricular ejection fraction; LVESVI, left ventricular end-systolic volume index; LVMI, left ventricular myocardium mass index; LVOTG, left ventricular outflow tract gradient; MWT, maximum wall thickness; NA, not applicable; and NT-proBNP, N-terminal pro B-type natriuretic peptide.

### Multivariate Analysis to Identify Predictors of the Presence of LGE

Multivariable binary logistic regression analysis was performed to identify potential predictors of the presence of LGE. Variables included in the multivariate analysis were described previously in statistical methods. As shown in Table 6, both log cTnI and MWT were independent predictors of the presence of LGE in patients with HOCM (OR = 3.193, P = 0.033; OR = 1.140, P <0.001, respectively), while log NT-proBNP was not. Additionally, resting LVOT gradient (OR = 0.979, P = 0.005), LVEF (OR = 0.866, P = 0.001), and usage of calcium antagonists (OR = 0.296, P = 0.017) were also independently associated with the presence of LGE.

**Table 6:** Multivariate logistic regression analysis for prediction of the presence of LGE.

Variable	OR	95% CI	P value
Calcium antagonists	0.296	0.109–0.803	0.017
Resting LVOTG	0.979	0.965–0.994	0.005
LVEF	0.866	0.793–0.945	0.001
Log cTnI	3.193	1.099–9.281	0.033
MWT	1.410	1.168–1.702	<0.001

CI indicates confidence interval; cTnI, cardiac troponin I; LGE, late gadolinium enhancement; LVEF, left ventricular ejection fraction; LVOTG, left ventricular outflow tract gradient; MWT, maximum wall thickness; and OR, odds ratio.

### Receiver Operating Characteristic Curve Analysis

**Fig 5:**
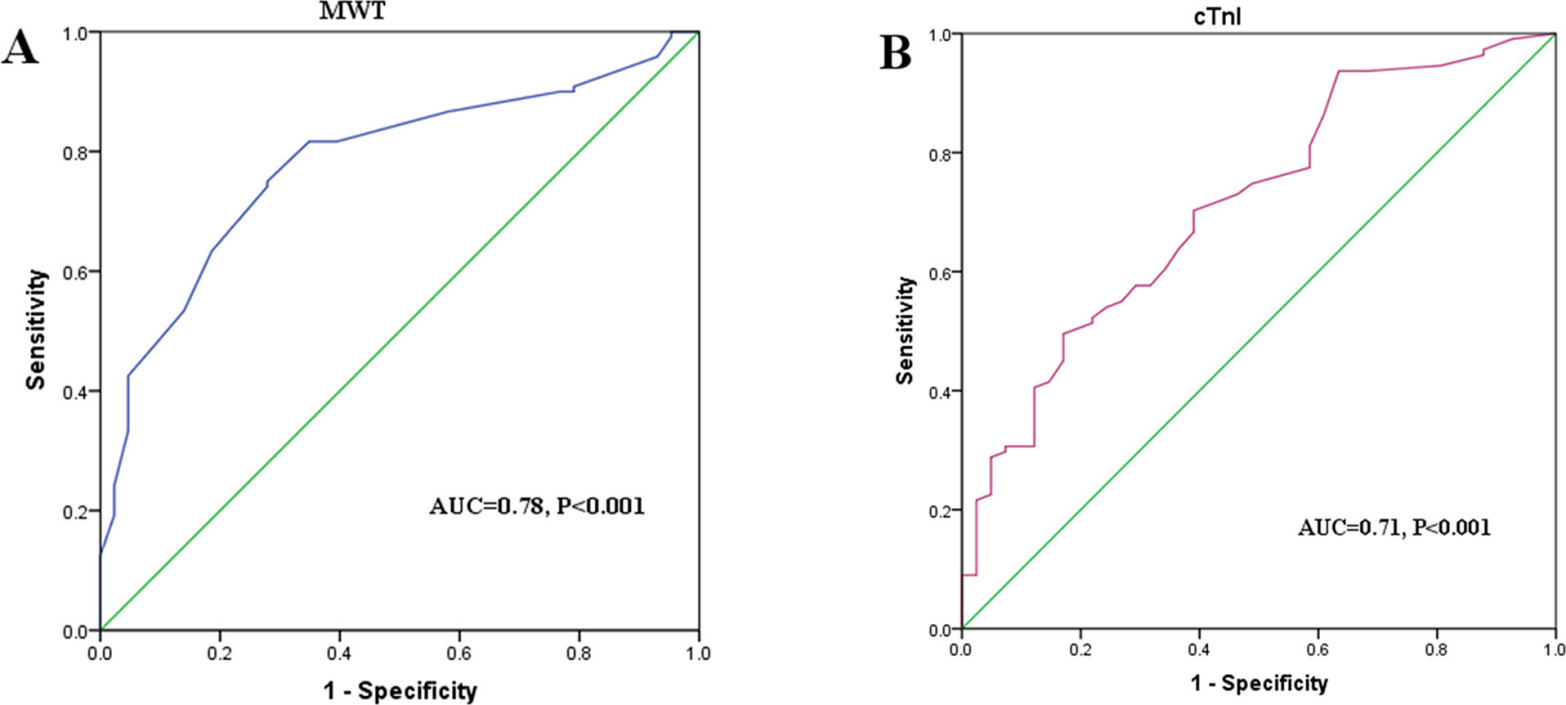
Receiver operating characteristic (ROC) curves of MWT (A) and cTnI (B) to predict the presence of LGE. AUC indicates area under ROC curve. Other abbreviations as in Figs 1 and 2.

Receiver operating characteristic (ROC) curve analysis was performed to evaluate the utility of MWT and cTnI as predictors for the presence of LGE (Fig 5). According to the ROC curve analysis, the optimal cut-off value of MWT to predict the presence of LGE was 21 mm, with a sensitivity of 75% and specificity of 72% (AUC = 0.78, 95% CI: 0.71–0.86, P <0.001); the optimal cut-off value of cTnI was 0.025 ng/ml, with 83% specificity and 50% sensitivity (AUC = 0.71, 95% CI: 0.62–0.80, P <0.001). Compared with MWT or cTnI alone, the combination of an MWT ≥21mm and cTnI ≥0.025 ng/ml yielded a high specificity of 95% to identify the presence of LGE, while the combined measurements of either MWT ≥21mm or cTnI ≥0.025ng/ml generated a high sensitivity of 88% (Table 7).

**Table 7:** Accuracy of MWT and cTnI in predicting the presence of LGE in patients with HOCM.

Variable	Sensitivity	Specificity	PPV	NPV
MWT ≥21 mm	75%	72%	88%	51%
cTNI ≥0.025 ng/ml	50%	83%	89%	38%
MWT ≥21 mm and cTNI ≥0.025 ng/ml	38%	95%	95%	35%
MWT ≥21 mm or cTNI ≥ 0.025 ng/ml	88%	60%	86%	64%

CTnI indicates cardiac troponin I; HOCM, hypertrophic obstructive cardiomyopathy; LGE, late gadolinium enhancement; MWT, maximum wall thickness; NPV, negative predictive value and PPV, positive predictive value.

## Discussion

To the best of our knowledge, this is the first study to investigate the association between circulating biomarkers and myocardial fibrosis determined by LGE-CMR in patients with HOCM. We found that peripheral concentrations of NT-proBNP and cTnI were significantly associated with the presence and severity of LGE in our HOCM cohort, on univariate analysis. After adjusting for other relevant variables in multivariate logistic regression analysis, serum cTnI together with MWT were independent predictors of the presence of LGE, whereas NT-proBNP was not. Furthermore, on ROC curve analysis, combined measurements of MWT ≥21 mm and/or cTnI ≥0.025 ng/ml showed good predictive values for the presence of LGE, with specificity of 95% or sensitivity of 88%. In addition, not taking calcium antagonists, decreased systolic function and reduced resting LVOT gradient also played a role in detecting myocardial fibrosis in these patients. Notably, the rates of LGE (74%) in the present study were high when compared to the literature (56% to 66%) [6, 7, 23], which may reflect differences in presentation, and the severity of HCM.

Multiple studies have been performed to determine serological markers of collagen metabolism in patients with HCM, however, their results are inconsistent [1, 24, 25]. In contrast, Kawasaki et al has demonstrated that the combination of high-sensitivity cardiac troponin T (hs-cTnT) and B-type natriuretic peptide (BNP) is useful in detecting myocardial fibrosis in patients with non-obstructive HCM [14]. This study sought to evaluate, in HOCM patients, the utility of NT-proBNP and cTnI, as well as CK-MB, hs-CRP, and big ET-1 to predict myocardial fibrosis as assessed noninvasively by LGE with CMR.

BNP is produced and released by ventricular and atrial cardiocytes, through multiple mechanisms [26–28]. Numerous studies have validated that circulating natriuretic peptides (including BNP and NT-proBNP) are elevated in patients with HCM, and significantly correlate with NYHA functional class, exercise capacity, maximal wall thickness, left ventricular mass index, LVOT gradient at rest, left atrial dimension, and diastolic function [8, 27, 29–32]. In addition, BNP and NT-proBNP predict long-term adverse events of HCM [9, 10, 12, 29]. However, there are scarce data about their relationships with cardiac fibrosis in patients with HCM. Paya et al suggested that patients with HCM with LGE had significantly higher levels of NT-proBNP than those without it [32]. Park et al reported that the level of NT-proBNP was positively correlated with the amount of LGE in patients with HCM [31]. Kawasaki et al showed that BNP level was not only higher in the LGE-positive group compared to the LGE-negative group, but also correlated well with the extent of LGE [14]. Similarly, the present study indicated that NT-proBNP levels were significantly higher in patients with LGE than in those without LGE, and were positively correlated with the extent of LGE as well as with maximum wall thickness, LVMI, resting LVOT gradient and left atrium diameter. However, NT-proBNP was not independently associated with the presence of LGE, after adjusting for other potential confounding factors in multivariate regression analysis.

The underlying mechanisms of correlations between elevated natriuretic peptides and cardiac fibrosis in HCM remain unclear. Possible explanations are as follows. First, the observed correlation between natriuretic peptides and fibrosis could be the consequence of their simultaneous positive relationships with the extent of left ventricular hypertrophy [6, 7]. In this study, NT-proBNP was shown to be an independent predictor of the presence of LGE (OR = 16.6, 95% CI: 1.8–155.3, P = 0.014) in multivariate regression analysis after controlling for other potential confounding factors except MWT and LVMI. Nonetheless, when MWT and LVMI were entered into the multivariate logistic regression analysis model, NT-proBNP was not associated with the presence of LGE anymore. Second, natriuretic peptides have a direct correlation with cardiac fibrosis apart from hypertrophy. Histologically, myocardial expression of BNP is significantly associated with the degree of fibrosis besides myofibrillar disarray and myocyte hypertrophy in endomyocardial biopsy specimens from patients with HCM [33]. In contrast to our findings, Park et al reported that LGE was significantly correlated with elevated NT-proBNP level, even after adjustment of MWT and LVMI [31]. In patients with HCM (including HOCM), myocardial fibrosis increases chamber stiffness and leads to passive diastolic dysfunction, eventually resulting in the elevation of natriuretic peptides [30, 34]. Third, BNP can be synthesized by cultured cardiac fibroblasts, inhibit fibrotic responses and activate the expression of matrix metalloproteinases [35]. Therefore, elevated peripheral natriuretic peptides may be partially released from cardiac fibroblasts, as a compensatory mechanism for cardiac fibrosis in HCM. The exact association between natriuretic peptides and cardiac fibrosis in HCM (including HOCM) remains to be elucidated.

Cardiac troponins, as specific and sensitive markers of myocardial damage, are well established diagnostic and prognostic biomarkers in patients with acute coronary syndrome [13]. Those elevated troponins predict mortality and combined adverse cardiovascular outcomes in patients with heart failure even in the absence of significant coronary stenosis [13]. Cardiac troponins, including cardiac troponin T and troponin I, are elevated in patients with HCM. Those elevated troponins are associated with MWT, LVMI, male gender, age, LV systolic dysfunction, LV diastolic dysfunction, left atrial diameter, LVOT gradient, atrial fibrillation and NYHA functional class [11–13, 36, 37]. Elevated baseline cardiac troponins are useful prognostic biomarkers of adverse cardiovascular events in HCM [12, 13]. The results of this study suggested that cTnI correlated positively with MWT, LVMI, LVEDVI, and LVESVI, and negatively with LVEF as described previously. Nonetheless, data concerning the relationship between fibrosis and cardiac troponins in HCM are rare and controversial. Moreno et al reported that hs-cTnT levels were significantly higher in HCM patients with LGE than in those without LGE [36]. Gommans et al found that the extent of fibrosis was higher in HCM patients with an elevated hs-cTnT.[37] In the study of Kawasaki et al, patients with LGE had higher levels of hs-cTnT compared to those without LGE [14]. Moreover, baseline hs-cTnT level was significantly correlated with the increase in the extent of LGE during 22 months of follow-up, although not with the initial extent of LGE. In contrast, no significant association was found between hs-cTnT concentrations and the prevalence of LGE in a study of 62 patients with HCM [11]. In the present study, the levels of cTnI were significantly elevated in the LGE-positive patients compared with the LGE-negative ones, and positively correlated with the extent of LGE. Specifically, we first indicated that serum cTnI was an independent predictor of the presence of LGE in patients with HOCM.

The potential explanations for correlations between myocardial fibrosis and cardiac troponins in HCM remain unresolved. Myocardial perfusion reserve was reduced in proportion to the magnitude of hypertrophy and fibrosis in patients with HCM [38]. Moreover, both myocardial fibrosis and circulating cardiac troponins are closely related to left ventricular hypertrophy in the present study and afore-mentioned HCM studies. Those findings together indicated that increased demand of the hypertrophied myocardium coupled with abnormalities of intramural coronary arterioles resulted in microvascular dysfunction, ischemia, myocyte necrosis, release of cardiac troponins and fibrosis [39]. Microvascular dysfunction was also found in non-hypertrophied myocardium of HCM patients [38]. The present study suggested that cTnI was an independent predictor of the presence of LGE even after adjustment of MWT and LVMI. Those observations suggested that abnormal intramural coronary arteries, independent of myocardial hypertrophy, lead to microvascular dysfunction, myocardial ischemia, release of cardiac troponins and subsequent fibrosis. Additionally, microvascular dysfunction and ischemia are important but not the sole cause of myocardial fibrosis in HCM [39]. In this respect, fibrosis may be secondary to specific genotypes, advanced disease, and the expression of a severe phenotype [39]. In addition to ischemia, myocyte disarray and reduced sarcomere responsiveness to stretch may potentiate myocyte injury and release of troponins into the circulation of patients with HCM [11].

In line with prior investigations in patients with HCM [6, 7, 23, 40], we also found that MWT and LVMI were univariately associated with the presence and extent of myocardial fibrosis. Furthermore, we demonstrated that MWT was independently related to the presence of LGE. In this study, combined measurements of MWT ≥21 mm and/or cTnI ≥0.025 ng/ml had excellent performance to predict the presence of LGE in HOCM patients, with a specificity of 95% or sensitivity of 88%. This is the first study showing that serum cTnI combined with MWT had good diagnostic value for detecting LGE in HOCM patients.

Consistent with previous reports [41, 42], this study showed a negative correlation between resting LVOT gradient and the extent of LGE. And as reported previously [7, 40, 42], we found that patients with LGE had lower LVEF and larger LVEDVI than those without LGE, and that the extent of LGE negatively correlated with LVEF. We speculate that the reverse correlation between the extent of LGE and LVOT gradient may be explained by the negative relationship between the extent of LGE and LVEF. That is, more fibrosis could result in less contractility, leading to a lower LVOT gradient even in HOCM patients with normal LVEF [41]. However, reduced resting LVOT gradient was a significant predictor of myocardial fibrosis independent of LVEF and LVEDVI. LV contractility is not the sole determinant of LVOT obstruction; redundant anterior mitral leaflet, abnormalities of subvalvular apparatus and load conditions also play a role [41].

In this study, compared to patients without LGE, those with LGE was less likely to take calcium antagonists. Patients taking calcium antagonists showed less severe LGE (15.9 ± 15.8% vs. 26.6 ± 19.0%, P = 0.001). Additionally, usage of calcium antagonists predicted less probable presence of LGE on multivariate regression analysis. The inverse relationship between calcium antagonists and the presence and extent of myocardial fibrosis in patients with HOCM haven’t been reported previously, further studies are needed to validate this phenomenon.

Similar to prior studies [40, 43], patients with LGE in the present cohort were significantly younger than those without LGE, and the extent of LGE inversely correlated with age at study entry. This study also suggested that the median duration from initial diagnosis of HOCM to study entry was longer in patients with LGE compared with those without LGE. All those findings concluded that an inverse relationship between age and myocardial fibrosis may partially account for more sudden death in young people with HCM [2]. However, age was not independently associated with LGE on multivariate regression analysis, large-scale follow-up studies are warranted to verify that hypothesis.

### Limitations

The present study had several limitations. First, this is a cross-sectional and single-center study conducted in a tertiary referral hospital, for which some inherent biases were unavoidable. Second, LGE was evaluated visually and indicated semi-quantitatively. However, this method was used frequently, and has been demonstrated to be feasible and reliable compared to the quantitative planimetry method [21, 22]. Third, LVOT gradient after provocation were not included in analyses, because only 50 of 163 patients in our cohort underwent provoked LVOT gradient measurements for those with resting LVOT gradient <50mmHg. Fourth, NT-proBNP and cTnI levels and LGE with CMR were measured at only one time point. Serial measurements of these parameters would be useful to further explore the dynamic relationships among them. At last, given that all of the enrolled subjects were patients with HOCM and without LV systolic dysfunction, our findings should be cautiously extrapolated to the general HCM population.

### Conclusions

As established biomarkers for cardiac overload and myocardial injury respectively, NT-proBNP is not, but serum cTnI is useful in identifying myocardial fibrosis, and combined measurements of serum cTnI and MWT further improve its value for predicting myocardial fibrosis in patients with HOCM and normal systolic function. If replicated, routine measurements of serum cTnI and MWT in daily clinical practice may become preferred substitute for LGE-CMR to assess myocardial fibrosis in HOCM, in consideration of their better accessibility and cost-effectiveness than LGE-CMR. Furthermore, gadolinium is not benign[44], particularly in advanced heart failure as renal dysfunction becomes more common, and in this regard, biomarkers associated with fibrosis may have additive value in risk prediction in HCM. In addition, not taking calcium antagonists, decreased left ventricular systolic function and reduced resting LVOT gradient also function for detecting myocardial fibrosis in these patients.

## Supporting Information

S1 Table (XLSX)

S2 Table (DOC)

S1 Text (DOC)

S2 Text (DOCX)
