# Supplementary material for: Predictive Values of N-Terminal Pro-B-Type Natriuretic Peptide and Cardiac Troponin I for Myocardial Fibrosis in Hypertrophic Obstructive Cardiomyopathy
Source: PLoS One. 2016 Jan 14;11(1):e0146572. doi: 10.1371/journal.pone.0146572 (PMC4713160; doi:10.1371/journal.pone.0146572)
Supplement: S2 Table — (DOC) [file pone.0146572.s002.doc]

STROBE Statement—Checklist of items that should be included in reports of ***cross-sectional studies***

|  | Item No | Recommendation |
| --- | --- | --- |
| **Title and abstract** | 1 | (*a*) Title in Page 1 |
|  |  | (*b*) Abstract in Page 2 |
| Introduction | | |
| Background/rationale | 2 | Page 3 |
| Objectives | 3 | Page 3, Paragraph 5 |
| Methods | | |
| Study design | 4 | Page 4, Paragraph 1 |
| Setting | 5 | Page 4, Paragraph 1 |
| Participants | 6 | Page 4, Paragraph 1 |
| Variables | 7 | Page 4, Paragraph 1 |
| Data sources/ measurement | 8* | Page 4, Paragraph 3 to Page 7, Paragraph 6 |
| Bias | 9 | Page 4, Paragraph 1; Page 4, Paragraph 2; |
| Study size | 10 | Page 4, Paragraph 1; Page 4, Paragraph 3 |
| Quantitative variables | 11 | Page 6, Paragraph 2 |
| Statistical methods | 12 | Page 7, Paragraph 7; Page 8, Paragraph 2 |
| Results | | |
| Participants | 13* | Page 8, Paragraph 3 |
| Descriptive data | 14* | Page 8, Paragraph 3; Page 10, Line 4; Page 11, Line 7 |
| Outcome data | 15* | None. |
| Main results | 16 | Page 17, Paragraph 1 |
| Other analyses | 17 | Page 18, Paragraph 2 |
| Discussion | | |
| Key results | 18 | Page 19, Paragraph 1 |
| Limitations | 19 | Page 23, Paragraph 2 |
| Interpretation | 20 | Page 19, Paragraph 2 to Page 23, Paragraph 1 |
| Generalisability | 21 | Page 23, Paragraph 3 |
| Other information | | |
| Funding | 22 | None |

*Give information separately for exposed and unexposed groups.

**Note:** An Explanation and Elaboration article discusses each checklist item and gives methodological background and published examples of transparent reporting. The STROBE checklist is best used in conjunction with this article (freely available on the Web sites of PLoS Medicine at http://www.plosmedicine.org/, Annals of Internal Medicine at http://www.annals.org/, and Epidemiology at http://www.epidem.com/). Information on the STROBE Initiative is available at www.strobe-statement.org.
