# Supplementary material for: Predictive Values of N-Terminal Pro-B-Type Natriuretic Peptide and Cardiac Troponin I for Myocardial Fibrosis in Hypertrophic Obstructive Cardiomyopathy
Source: PLoS One. 2016 Jan 14;11(1):e0146572. doi: 10.1371/journal.pone.0146572 (PMC4713160; doi:10.1371/journal.pone.0146572)
Supplement: S1 Text — (DOC) [file pone.0146572.s003.doc]

Dear editor,

Thank you very much for evaluating my manuscript entitled "Predictive Values of N-terminal Pro-B-Type Natriuretic Peptide and Cardiac Troponin I for Myocardial Fibrosis in Hypertrophic Obstructive Cardiomyopathy" to PLOS ONE. The assigned manuscript number is PONE-D-15-33682.

I've been asked to clarify some issues for my manuscript and documents. The following are my response:

1. According to the legislation of China and the principle of Fuwai Hospital, retrospective data collection does not require approval of an ethics committee.

2. A	STROBE checklist has been reviewed, and we could assure that our manuscript is updated with all of the information recommended by STROBE.

3. This was a retrospective study in which we analyzed medical record data.

4. The medical records were anonymized and de-identified before access and analysis. No author interacted with patients or had access to patients' identifying information. All of our patients had signed consent to having their medical records and samples used in the future research, during their medical care in our hospital. However, informed consent to specifically participating in this study was exempted because of the retrospective nature of the current study.

Moreover, we have made corresponding amendments in our manuscript according to your requests.

Thank you very much for your careful attention to our submission.

Sincerely yours,

Shubin Qiao

Corresponding author:

Email: qsbfw@sina.com (SQ).
